# Built environment and diabetes

**DOI:** 10.4103/0973-3930.62594

**Published:** 2010

**Authors:** Sudhir Kumar Pasala, Allam Appa Rao, G. R. Sridhar

**Affiliations:** Department of Architecture, Andhra University College of Engineering (Autonomous), Visakhapatnam, India; 1 JNTU, Kakinada, India; 2 Endocrine and Diabetes Center, Visakhapatnam, India

**Keywords:** Diabetes, lifestyle, stress, sedentary, sleep, relaxation, obesity

## Abstract

Development of type 2 diabetes mellitus is influenced by built environment, which is, ‘the environments that are modified by humans, including homes, schools, workplaces, highways, urban sprawls, accessibility to amenities, leisure, and pollution.’ Built environment contributes to diabetes through access to physical activity and through stress, by affecting the sleep cycle. With globalization, there is a possibility that western environmental models may be replicated in developing countries such as India, where the underlying genetic predisposition makes them particularly susceptible to diabetes. Here we review published information on the relationship between built environment and diabetes, so that appropriate modifications can be incorporated to reduce the risk of developing diabetes mellitus.

## Introduction

It is evident that diabetes mellitus, an increasingly prevalent condition, must be approached in an appropriate, cost-effective, and culturally relevant manner.[1–3] The environmental and lifestyle modifications that are taking place, primarily in developing countries, are appropriate factors in the prevention of diabetes.[4] The living environment significantly contributes to the lifestyle and habits of its inhabitants, including opportunities for exercise, food, rest, relaxation, and sleep. In developed countries, the urban sprawl separates the working from living spaces geographically, leading to dependence on vehicles for meeting most of the necessities of life. Studies have shown that the urban sprawl was associated with obesity and diabetes mellitus.

Built environment is the term used to describe ‘environments that are modified by humans, including homes, schools, workplaces, highways, urban sprawl, accessibility to amenities, leisure, and pollution,’ influencing peoples behavior, leading to a sedentary lifestyle.

That physical exercise can prevent the onset of diabetes is true in India as much as anywhere else. Also, as stress and lack of sleep can lead to obesity and insulin resistance, it is appropriate to evaluate the influence of ‘built environment’ as a contributor to the diabetes epidemic.[5–7]

## Built Environment – Physical Inactivity – Obesity and Diabetes

### Urban Sprawl

Urban planning practices in developing countries, for example, the urban grid and single-use zoning, may contribute to the epidemics of obesity and diabetes. The National Health Interview Survey, NHIS (1997 – 1998) found that men living in cities were more likely to be obese (39.4%) than suburban men (35.5%). Similarly, in women 20.6% were obese versus 19.1% in the urban and rural areas, respectively.[8] Increased levels of sprawl were associated with increased obesity, due to decreased physical activity.[9,10] Furthermore, factors such as cul-de-sacs, lack of parks, high-speed traffic, and automobile-focused transport could discourage activity and ultimately increased the risk of obesity.[11] Neighborhoods consisting exclusively of housing seemed to dampen physical activity. ‘The broken window syndrome — neighborhoods with broken windows and dilapidated housing,’ had a social effect of abandonment, encouraged crime, and isolated residents, reducing trust in the neighborhood for social interactions.[12] These factors of neighborhood quality were linked with reduced walking, physical activity, and recreation in public.

**Figure 1:**
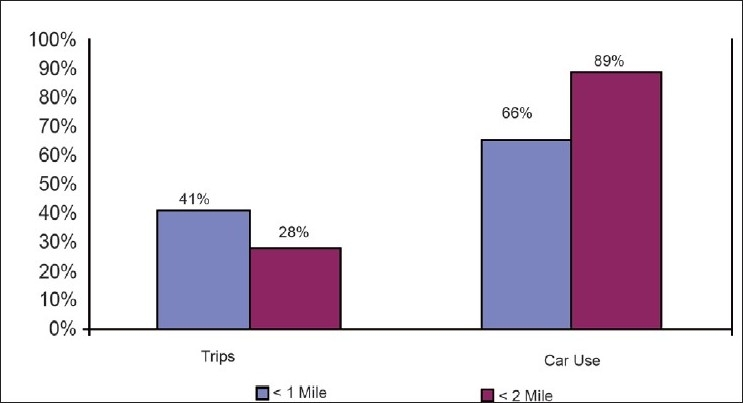
Comparison of car use and distance

The physical features of the urban environment influence the manner in which city residents live and work, and have a direct impact on mobility and social interactions. In a study, carried out in 2001, in the metropolitan areas of the United States, 41% of all trips were shorter than two miles; 28% were shorter than one mile.[13] Bicycling could easily cover distances of up to two miles, and most people could walk at least a mile.[14] Yet Americans use their cars for 66% of all trips up to a mile long and for 89% of all trips between one and two miles long [Figure 1].[13] Road construction and automobile dependency have also been associated with community severance (i.e., reduced access to local amenities and disruption of social networks caused by a physical barrier running through the community).[15–19] Higher dependence on motor vehicles in urban areas has resulted in higher levels of congestion[20] and increased motor and pedestrian injuries and deaths.[21]

### Vehicular

A study in California explored the association of distances with an indicator of transportation data (Vehicle Miles of Travel, VMT) at the county level and relation to obesity and physical inactivity.[22] Data from the California Health Interview Survey, CHIS-2001, the US 2000 Census, and the California Department of Transportation were merged to examine the ecological correlations between VMT, population density, commute time, and county indicators of obesity (measured by Body Mass Index, BMI) and physical inactivity, based on self-reported behaviors. Statewide obesity prevalence ranged from 11.2 to 28.5% by county, physical inactivity ranged from 13.4 to 35.7%, and daily VMT ranged from 3.3 million to 183.8 million per county. By rank bivariate correlation, obesity and physical inactivity were significantly associated (*P* < 0.01). Furthermore, by rank analysis of variance, the highest mean rank obesity was associated with the highest rank of VMT (*P* < 0.01). Similar rank patterns were observed among obesity, physical inactivity, and commute time. Associations between VMT and physical inactivity were examined, but failed to reach statistical significance. This analysis adds to the growing evidence supporting the association between VMT and obesity.

### Pedestrian

Pedestrians perceive trip making mainly in spatial terms; distance is a more suitable measure of proximity, others being impedance / controlling factors.[23] Impedance factors are those that pose potential barriers to walking or bicycling.[24] Besides distance, impedance / controlling factors include steep slopes, nightfall, precipitation, and less secure environs. Environmental factors contribute to low levels of lifestyle physical activity[25] that are assessed by the walking proximity from home to various types of facilities, with responses ranging from a one-to-five minute walking distance (coded as 5) to 30-minute walking distance (coded as 1).[26] Higher scores on mixed land use indicate closer average proximity. Furthermore, residents in high walkability neighborhoods perceived their neighborhoods as having higher residential density, mixed land use, street connectivity, aesthetics, and pedestrian / automobile traffic safety than did residents of low walkability neighborhoods.

Another household activity data from the San Francisco region was used for factor analysis to represent the urban design and land-use diversity dimensions of built environments. Combining proximity factor scores with control variables discrete-choice models were estimated. Built-environment factors exerted far weaker, although not inconsequential, influences on walking and bicycling than control variables. Stronger evidence on the importance of urban landscapes in shaping foot and bicycle travel is expressed to forge an effective alliance against the car-dependent urban sprawl.[27]

European countries with the highest levels of walking and cycling have much lower rates of obesity, diabetes, and hypertension than the United States.[28,29] The Netherlands, Denmark, and Sweden, for example, have obesity rates only a third of the American rate, while Germany's rate is only half as high.[29] Moreover, the average healthy life expectancies in those four European countries are 2.5 to 4.4 years longer than in the United States,[28] although their per capita health expenditures are only half of those in the United States.[30] Regular physical activity fosters good physical and mental health.[31] One needs to accumulate only 30 minutes per day, five days per week, of moderately intense physical activity, such as brisk walking.[32]

### Lifestyle and social issues

There are social, health, and economic consequences to isolated and sedentary lifestyles. Higher rates of television viewing, increased computer usage, little contact with neighbors, and crime, leads to lack of social network contributing to obesity, cardiovascular disease, mental health problems, and increased rates of mortality.[33–36]

## Built Environment – Physical Activity – Experiences of the Western world

The Center for Disease Control and Prevention conducted a workshop to identify the best practices for designing new communities and revitalizing old ones in ways that promote physical and mental health. The workshop participants' areas of expertise included physical activity, injury prevention, air pollution, water quality, urban planning, transportation, architecture, land use, mental health, social capital, housing, and social marketing. Community characteristics — proximity of recreation; facilities; street design; housing density; and provision for safe pedestrian, bicycle, and wheelchair use, played a significant role in promoting or discouraging physical activity.[37,38] NHIS found that certain features of the built environment, such as, the presence of sidewalks, streetlights, interconnectivity of streets, population density, and mix use, appear to encourage physical activity and thus reduce the risk of obesity and related health problems. Studies find that people who live close to parks are more likely to use them and to be physically active than those who live further away from them.[39] Neighborhoods with a mixture of land use types, commercial, industrial, residential, and offices, also appear to promote physical activity.[39]

## Neighborhood resources

### Convenient places and awareness of places

Three categories of convenience were created based on time and mode of travel to the place: (1) less than 10 minutes walking, (2) less than 10 minutes non-walking, and (3) 10 minutes or greater regardless of mode. An estimated 91.8% (with 95% confidence interval) of Georgians had a place where they felt safe, walking for exercise or recreation indicating the value of convenient places for activity.[38–41] The findings confirmed the association between awareness of places and physical activity practices,[42] and also that neighborhood streets, sidewalks, and public parks were the most commonly reported safe and convenient places for walking.[41] Noting the association between self-reported convenience (time and mode of getting to place) and physical activity in the survey, the availability[38–41] and awareness[42] of safe and convenient places (such as trails, parks, sidewalks, and treadmills) conducive to physical activity are associated with higher levels of physical activity.

## Safe Routes and Proximity

About one-third of the children in the US take a bus to school and half are driven in a private vehicle.[43] To increase the proportion of children walking and biking to school, a program was incorporated into the National Health Objectives for 2010.[25] A multi-pronged approach was used to identify and create safe routes to schools, to increase the number of Marin County children walking and biking to school, and to decrease the number of school trips made by private vehicles. The student transportation surveys revealed an increase in walking, biking, and carpooling in the participating public schools. There was a 64% increase in the number of children walking, a 114% increase in the number of students biking, 91% increase in the number of students carpooling, and a 39% decrease in the number of children arriving by private cars, carrying only one student. Two private schools, drawn from larger geographical areas, recorded only a modest increase in walking (1%) and carpooling (5%) and a small decrease in biking (− 1%) and ‘drive-alone’ transport (− 4%) showing evidence of proximity playing a major role.[44]

## Bicycle streets for safe routes

Traffic calming limits the speeds (30 KMPH or Less) of motor vehicle traffic, both by law and through physical barriers such as raised intersections and crosswalks, traffic circles, road narrowing, zigzag routes, curves, speed humps, and artificial dead ends created by mid-block street closures facilitating safe bicycling.[14] Also, Provision for ‘bicycle streets,’ where cars are permitted, but cyclists have strict right-of-way over the entire breadth of the roadway is another promising element for safe bicycling.

## Social capital and place making

To invigorate neighborhood stewardship, the Sunnyside neighborhood in the Portland community organized and created a public gathering place by painting a gigantic sunflower in the middle of an intersection and installed several interactive art features.

In April 2003, of 507 pedestrians observed to pass through the intersection, 164 (32%) interacted with the piazza, compared to 7% at a similar, unimproved intersection. Walking and biking increased, as people went out of their way to enjoy the richness of the urban experience at the Sunnyside Piazza. A perceived sense of community was documented as part of a cross-sectional survey that systematically sampled participants within a two-block radius of the Sunnyside Piazza and participants of an adjacent neighborhood with a similar demographic profile (participation rate = 60%). Of 97 Sunnyside Piazza residents interviewed, 65% rated their neighborhood as an excellent place to live, compared with 35% of 147 residents interviewed from the adjacent site.[45]

## Community gardens

Community gardens enhance nutrition and physical activity and promote the role of public health in improving the quality of life. California Healthy Cities and Communities (CHCC) has funded community-based nutrition and physical activity programs in several cities incorporating local leadership and resources, volunteers and community partners, and skill-building opportunities for participants. In one of such program with the Center for Civic Partnerships / Public Health Institute, CHCC launched a school gardening program with nutrition and physical activity education in West Hollywood. The participants (n = 338) increased the number of physical activity sessions from 4.9 to 5.2 times per week (6%) and increased consumption of fruits and vegetables from 3.44 to 3.78 servings per day (10%). In the city of San Bernardino, the number of students that began gardening at home after participating in the school gardening program increased from 62 to 75 (20%).[46]

## Awareness and education

In a recent study, the increasing awareness and empowerment of the community was shown in the prevention of diabetes and other non-communicable disorders.[47] Mass awareness programs like public lectures, video clippings, and distribution of educational pamphlets were carried out in a residential colony in Chennai for three years. A follow up study, seven years later, showed a 277% increase in the proportion of walkers. The proportion of individuals who, exercised increased from 14.2 to 58.7%.[48] The colony residents motivated by the awareness programs constructed a park with the help of the civic authorities, which is now being used regularly not only by the residents, but also by the neighboring colonies.

## Conclusion

Diabetes is due to strong genetic factors coupled with urbanization and lifestyle changes. Many of the adverse environmental factors are modifiable. Prevention of Type 2 diabetes will require measures to promote physical activity and stress reduction measures in the built environment they live in, and to reduce obesity in adults and children.

Built environment may be defined as ‘the environments that are modified by humans, including homes, schools, workplaces, highways, urban sprawls, accessibility to amenities, leisure, and pollution, including public policy and political action.’

The first step is to better understand the elements of the built environment that promote health. Some environments encourage walking, biking, and social interaction more than others do. Overall, there is still much to learn about the effects of the built environment on health. To address the multitude of questions, coordination with experts in many fields is required. Most importantly, we need to look to the future rather than the past. We cannot go back in time and become hunters or gatherers for physical activity, to get rid of obesity / diabetes, and we are not going to get rid of the technological changes – we must move forward. The current generation now faces its challenges of the impact of built environment on health, especially the chronic diseases that are rampant in urban areas, to build future communities that promote physical and mental health.
